# The Creation of a Pediatric Surgical Checklist for Adult Providers

**DOI:** 10.21203/rs.3.rs-3269257/v1

**Published:** 2023-09-13

**Authors:** Diana Rapolti, Phyllis Kisa, Martin Situma, Elsa Nico, Thom Lobe, Thomas Sims, Doruk Ozgediz, Greg Klazura

**Affiliations:** University of Illinois Hospital and Health Sciences System; Mulago National Referral Hospital; Mulago National Referral Hospital; University of Illinois Hospital and Health Sciences System; University of Illinois Hospital and Health Sciences System; University of Illinois Hospital and Health Sciences System; University of California, San Francisco; University of Illinois Hospital and Health Sciences System

## Abstract

**Purpose:**

To address the need for a pediatric surgical checklist for adult providers.

**Background:**

Pediatric surgery is unique due to the specific needs and many tasks that are employed in the care of adults require accommodations for children. There are some resources for adult surgeons to perform safe pediatric surgery and to assist such surgeons in pediatric emergencies, we created a straightforward checklist based on current literature. We propose a surgical checklist as the value of surgical checklists has been validated through research in a variety of applications,

**Methods:**

Literature review on PubMed to gather information on current resources for pediatric surgery, all papers on surgical checklists describing their outcomes as of October 2022 were included to prevent a biased overview of the existing literature. Interviews with multiple pediatric surgeons were conducted for the creation of a checklist that is relevant to the field and has limited bias.

**Results:**

42 papers with 8529061 total participants were included. The positive impact of checklists was highlighted throughout the literature in terms of outcomes, financial cost and team relationship. Certain care checkpoints emerged as vital checklist items: antibiotic administration, anesthetic considerations, intraoperative hemodynamics and postoperative resuscitation. The result was the creation of a checklist that is not substitutive for existing WHO surgery checklists but additive for adult surgeons who must operate on children in emergencies.

**Conclusion:**

The outcomes measured throughout the literature are varied and thus provide both a nuanced view of a variety of factors that must be taken into account and are limited in the amount of evidence for each outcome. We hope to implement the checklist developed to create a standard of care for pediatric surgery performed in low resource settings by adult surgeons and further evaluate its impact on emergency pediatric surgery outcomes.

**Funding:**

Fulbright Fogarty Fellowship, GHES NIH FIC D43 TW010540

## Introduction

Surgery is a vital element of healthcare with the potential to cause serious harm when performed in an unsafe manner. A recent World Health Organization (WHO) survey estimates complications occur in about a quarter of surgical patients. ^1^ A large portion of cases in which those serious complications occur are preventable and are related to non-technical skills^2^.

To reduce adverse events such as these, the WHO developed a Surgical Safety Checklist (SSC) in 2008. The checklist comprises three phases and 19 items addressing a variety of perioperative safety measures. The mechanism for improving surgical safety is two-fold: through direct action it standardizes what the team does for every procedure and indirectly it promotes a culture of safety in the operating room. ^3^ This checklist and others inspired by it have been implemented worldwide with a variety of results.

There is heterogeneity in terms of outcomes studied, however, overall multiple papers suggest that checklists are beneficial: decreasing cost, complications and mortality while improving teamwork and communication. The current literature also highlights the importance of staff perception of SSC with staff attitudes towards SSC affecting how often it is utilized and how it is altered to better adapt to their context. ^4^

As the focus of research on surgical checklists has increasingly shifted to include more tailored checklists, their application in pediatric surgery remains largely unexplored. This gap in the literature is of particular importance as it could assist adult surgeons who often must operate on children in emergency circumstances. This is especially true in rural settings and in low- and middle-income countries (LMIC) like Uganda where general surgeons perform the majority of general pediatric surgeries. ^5,6^ In the USA as many as 40% of all pediatric inpatient surgical procedures are performed in adult hospitals.^7^ Furthermore, children are far more complex than just smaller adults yet the WHO SSC does not consider and fully address the intricacies of pediatric surgery. Given the potential worldwide impact of a pediatric surgery checklist for adult general surgeons, we reviewed existing literature on surgical checklists and created a fundamental checklist that surgeons in a variety of resource settings can utilize. Resuscitation, consent, pain control and postoperative care for pediatric patients all require special consideration when the adult surgeon is called to operate on a child. Low and high resource settings may contract or expand this checklist based on their resources and needs. This essential checklist of considerations serves as a guide for adult surgeons needing to operate on children.

## Methods

The literature review was conducted using PubMed and the University of Illinois library. Papers with text words and subject headings including “surgical checklist” were identified and reviewed. Reference lists from papers identified in the PubMed search were also reviewed and included when appropriate.

Pediatric surgeons at the University of Illinois at Chicago (UIC) Division of Pediatric Surgery and the Paediatric Surgical Foundation of Uganda (PSFU) identified checklist items that they felt were both vital and specific to pediatric general surgery surgery. Dr. Phyllis Kisa from Mulago National Referral Hospital and Dr. Martin Situma from Mbarara Regional Referral Hospital in Uganda participated in the creation of this checklist and provided valuable insight into its potential real world application in LMICs from their own clinical experience. Dr. Lobe, Dr. Sims, and Dr. Rojnica from the UIC Division of Pediatric Surgery also helped create checklist items they deemed essential for adult surgeons performing pediatric surgery in their setting.

We then integrated checklist items from UIC and PSFU with key findings from our comprehensive literature review to create three age appropriate, contextually adaptable checklists for pediatric surgery.

## Results

The majority of papers reviewed employed the WHO SSC and its specific adaptations. (Table 1).^8^ No existing pediatric surgery checklists were identified in our review of the literature.

### Checklist Effect on Complications and Mortality

Checklists have been shown to reduce postoperative complications, including SSI and mortality. The WHO SSC specifically targets mortality ^9,10^, SSI ^11^, pneumonia ^12^, return to the operating room ^13^, urinary tract infection, intubation, and sepsis.^14^ The WHO SSC has shown positive changes in regards to all of these targets.^15,16^ Thromboembolism (DVT), however, was not a target. Investigations have shown that although the WHO SSC does affect measures like mortality and SSI it does not affect postoperative measures of safety and quality that are not targeted, like DVT.^12^ Maternal sepsis rates were also found to be reduced with the use of the WHO SSC with adherence negatively correlating with sepsis rates.^17^ Further, there is evidence that intraoperative blood loss and incidence of postoperative intestinal fistula formation was lower with the SSC.^18^ Impact on mortality and SSI has been suggested to be more significant in emergency settings in low and middle income countries. ^19,20^

### Checklist Effect on Teamwork, Communication, and Culture of Safety

The impact of SSC implementation on teamwork and communication was almost unanimously positive across all the studies. After SSC intervention, Molina et al (2016) reported improvements in team discussions, physician receptiveness to quality improvements, and overall communication by 15%, 9%, and 11.9%, respectively.^21^ Zingiryan et al (2017) reported improved communication in 76.4% of participants.^22^ White et al (2018) reported improved teamwork and communication in 91% and 89% of participants.^23^ Tan et al (2021) reported improved communication in 85% of participants.^24^ One study, however, stood out as an exception; it demonstrated that while nurses and anesthesiologists experienced significantly fewer communication failures, surgeons found no difference in communication with SSC use.^25^ Despite this outlier, other studies note that although nursing staff involvement is especially important for compliance, support from surgeons is also critical.^26–30^ Notably, safety culture also improved and was likely correlated with fidelity to a checklist.^31^ However, that fidelity appeared to be compromised when staff perceived the checklists as “add ons”.^32^

### Checklist Financial Impact

Few studies investigated the financial impact of SSC; however, those that did noted SSC implementation was a cost effective health intervention. Checklist implementation costs, length/cost of hospital stay, blood transfusion, antibiotics used in the OR, the cost of OR time, and the economic gain from additional years of life expectancy were considered in studies that did evaluate the financial impact of SSC. In their single-center assessment, Healey et al (2020) determined that for every 100 admissions the SSC cost $900 to implement but saved $55,899 overall. ^33^ Yu et al (2020) discovered significantly lower hospitalization costs while Haugen et al (2019) witnessed a 40% reduction in blood transfusion costs with implementation of the SSC. ^18,34^ The SSC incremental cost-effectiveness ratio (ICER) for one year of life loss averted was $31–118 and for every $1 spent on checklist implementation $9–62 was saved. ^35^

### Checklist Creation

Research indicates that checklists perform best when they are targeted, simple, and contextually appropriate.^36,37^ Almeida et al (2021) analyzed all surgeries performed at their hospital or in their country to gain a more comprehensive view of SSC impact.^11^ Their findings highlighted the need for a tailored checklist.^11,37^ Others found that involving hospital staff in checklist creation helps create a contextually appropriate checklist.^36^

Although contextually appropriate checklists are best, this of course has its limits. A checklist made for just one setting has more limited utility. With this in mind, using findings from our literature review, and receiving input from pediatric surgeons in HICs and LMICs we created three age specific, adaptable, general pediatric surgery checklists: Neonatal, Infant, and Toddler/Child.

These checklists have room for contextually-appropriate modifications depending on the operation and resources available. Below is the Neonatal checklist as an example. All three checklists are also located in the appendix.

We also determined that there are important points on neonatal, infant, and child physiology that the provider should be aware of prior to following the checklist, administering resuscitation, and delivering anesthesia (Appendix 2). This information complements the checklists and should serve as a reference for providers who care for the sick surgical child. Broselow Tape is an additional reference that can be used to estimate appropriate tube sizes, medication doses, and defibrillator shock doses but its accuracy has been shown to be limited in recent studies.^38^

## Discussion

Research focusing on a variety of surgical subspecialties including general surgery, neurosurgery, plastic surgery, otolaryngology, orthopedics have shown the positive impact of checklists on clinical outcomes. ^3,9–11,13,14,17–20,33,34,45–64^ The evidence for checklist impact overall, however, is quite heterogeneous in terms of outcomes studied and the estimated magnitude of the impact of the checklist. Table 1 attached in the appendix displays the current literature on checklists and shows this variation in existing literature. Nevertheless, the consensus impact of SSC remains generally positive.

One challenge in evaluating checklist implementation is that different research groups have investigated different post-surgical outcomes. Studies have focused on surgical site infections, in-hospital mortality, overall mortality, blood loss, reoperation, embolism and other adverse outcomes. Although this complicates the overall picture when comparing studies and some types of post-surgical outcome have limited evidence, it also provides a more complete description of the many elements that might be improved through the use of the SSC.

Another critical element of SSC use explored throughout the literature is the variability in adherence and attitudes towards SSC and their impact on clinical outcomes. Overall, staff attitudes are critical for utilization compliance.^26^ This perhaps suggests that regular training and education on the purpose of SSC are important for engagement of the team.^65^ Training should specifically target collaboration with the surgical team since their cooperation is the most commonly cited obstacle to successful implementation.^28,66,67^ These trainings should also have implementation procedures which consider previous experiences and feedback in order to most effectively create a culture of safety.^68,69^ When implementing a SSC it is also important to consider the burden on a workforce in under-resourced settings that is often stretched too thin. Ultimately, SSC’s should not create more work but rather decrease workload through improved patient outcomes.

Although a majority of providers have positive opinions of surgical checklists, there remains a gap in knowledge about their use.^67^ In order to bridge this gap there is some evidence that digital SSC displayed on OR monitors increases engagement and accessibility.^70^ Many settings, however, do not have an OR computer monitor and efforts to bridge this gap must be made elsewhere.^29^ As with the consideration of not creating more work it is vital to adapt these findings to the local resources as the goal of the SSC is to standardize surgical care and provide guidance

Towards the goal of providing standardized guidance for pediatric patients, Ugandan pediatric surgeons also developed the Pediatric Emergency Surgery Course (PESC). It is a three day course targeted at rural general surgeons and healthcare providers^71^. Similar to this checklist the course aims to improve resuscitation and referral patterns for complex surgical conditions such as high anorectal malformations. It also aims to increase provider confidence treating less complex conditions such as pyloric stenosis. The course has been reviewed favorably, demonstrating statistically significant improvements in provider knowledge^71^. In the future, checklist implementation could coincide with educational interventions such as the PESC. Not only should future work coincide with contextually appropriate training but also investigations and feedback should be gathered from providers who use the checklist so improvements can be made.

As stakeholders improve surgical outcomes and safety locally and globally special consideration should be given to pediatric surgery checklists. Surgical disease represents roughly 28% of the world’s burden of disease.^72^ This burden disproportionately affects children in LMICs; up to 85% of children in LMICs have a surgically-treatable condition by age 15.^73,74^ The lack of a pediatric surgery checklist for any setting further demonstrates the need and potential benefits of a pediatric surgery checklist that can be adapted for different resource levels. Further research on the topic is necessary especially regarding the differences between implementing such a checklist in HIC and LMIC hospitals. As the first checklist seeking to inform surgical care on children for providers without significant specialized training and in urgent, and often resource limited settings, it is important to evaluate its implementation and effectiveness for adult general surgeons.

Although this checklist had input from pediatric surgeons in HICs and LMICs, UIC, Mulago and Mbarara were the only institutions represented in its creation. Our pediatric checklist seeks to integrate as much knowledge from the pediatric surgeons involved in its creation, however it is limited to their experiences and the resources available in their institutions. We acknowledge that other checklists exist already and some may argue against the utility of this checklist and its specificity to pediatric general surgery. It has however been shown throughout this paper that specific checklists have a role to play in different surgical subspecialties, thus supporting our work in the creation of this framework for pediatric general surgery. Although there exist books with pediatric surgery considerations, a concise checklist indicating clear actions that are important for providers is necessary for settings with limited resources. Countries such as Uganda with few pediatric surgeons, general surgeons are required to fill the gaps and provide care to children without a clear standard of care. The next step for standardizing pediatric surgical care in resource limited settings would be evaluating the effectiveness of our pediatric surgery checklist in practice by adult general surgeons in a variety of settings in HICs and LMICs.

## Conclusion

The benefits of surgical checklists are far reaching: improved teamwork, communication, clinical outcomes, and patient safety all while saving hospitals and patients money. Keeping in mind that checklists are most effective when they are tailored to the context and the patient, we created three general pediatric surgery checklists that can be adapted to different settings based on resource availability and specific needs. This is the first set of checklists developed specifically for pediatric surgery and providers should carefully weigh their benefits as they consider how to appropriately use them in their practice. This peer reviewed checklist steeped in robust literature review is a critical first step in further standardization of pediatric surgical care and highlights the most important considerations in pediatric surgery in a way that is accessible and concise for general surgeons to use in their practice.

## Figures and Tables

**Table 1:** Summary of literature review on current research regarding surgical checklists and their impacts. Location of study and checklist used were highlighted.

Citations	N	Study location	Type of Surgery	Checklist used	Impact of checklist
Moore et al. 2022^9^	Before checklist: 9475 18 months after: 10589 9 years after 57577	Auckland City Hospital, New Zealand	Majority MSK: 27%, GI: 25%, Urinary: 12% Neurological: 11%, Cardiovascular: 10%, Derm and plastics: 6%, Male genital organs: 5%, Other: 5%.	WHO surgical safety checklist	Mean number of days alive and out of hospital after checklist implementation was 1.0 (0.4–1.6) days longer than in the cohort preceding implementation. 90-day mortality was 4% before and 3% after SSC, not statistically significant.
Healey et al. 2022^33^	3702, control: 1398, checklist: 2304	Norway	Orthopedic: 61.5%, thoracic: 18.5%, Neuro: 20%. 53.1% elective and 46.9% emergency. General anesthesia in 59.3% and regional in 40.7%	WHO surgical safety checklist	Implementation of the WHO checklist resulted in an additional 5.9 complication-free admissions per 100 admissions
Wurdeman et al 2022^17^	1341	Tanzania, 20 facilities in Lake Zone	Cesarian section	WHO surgical safety checklist	Higher SSC adherence was associated with lower rates of maternal sepsis: <25% adherence: 5.0%; >75% adherence: 0.7%. Adjusted OR of 0.17 per percentage point increase in SSC adherence. Wound class significantly associated with maternal sepsis: Clean-Contaminated 3.7% vs Contaminated/Dirty 20%
de Almeida et al 2021.^11^	1025, 486 before implementation and 539 after implementation	Brazil	Elective and acute surgery	WHO surgical safety checklist	Significant reduction in SSI, OR 0.33. Reduction of SSI for contaminated and infected wounds, and for those in whom antimicrobial prophylaxis was discontinued < 48h. Reduced antimicrobial resistance. Reduction in hospital deaths 6.4–3.2%.
Ngonzi et al 2021^46^	678 (pre-intervention: 200, intervention: 230, post-intervention: 248)	Uganda, referral hospital	Cesarian section	WHO surgical safety checklist	Pre-intervention antibiotic use was 18% versus 90% in intervention phase and 84% post-intervention phase. SSI rate in the pre-intervention phase was 15% versus 7% in intervention phase and 11% post-intervention
Storesund et al 2020^13^	checklist: 9009, controls: 9678	Norway, tertiary hospital	Control: 16.3%, neurosurgery, 46.9% orthopedics, 36.7% gynecology. Regional: 33.7%, general: 66.3%. Checklist: 37.2%, neurosurgery, 51.0% orthopedics, 11.8% gynecology. Regional: 35.1%, general: 64.9%	Adapted WHO surgical safety checklist (preoperative and postoperative checklists)	Reductions in complications with OR 0.70 and emergency reoperations with OR 0.42. Reduced readmissions, OR 0.32. No changes in mortality or LOS. Overall increased complications for parallel controls.
Yu et al. 2020^18^	1072 (checklist: 556, control: 526)	China, tertiary referral hospital	Surgery for gastric cancer	Perioperative Safety Checklist for Gastric Cancer (designed by researchers)	Reduction in postop intestinal fístula formation, unplanned secondary surgery, and total hospitalization expenses. Intraoperative blood loss in the complete and partial implementation groups signifícantly lower than in no implementation group, hospitalization cost showed an opposite trend.
Chhabra et al. 2019^45^	Control: 250, Checklist: 250	India	Urology, breast, gall bladder, hernia stoma reversal, anorectal malformations, other abdominal and thyroid surgeries	WHO surgical safety checklist	Major wound disruption: 10.8% control and 5.2% checklist group. Control group 29.2% SSI vs 13.6% in checklist group. 2% patients in the control group developed sepsis while no patients in the checklist group did.
de Jager et al 2019^49^	21306	Australia, tertiary hospital	Variety of procedures. Both general and regional anesthesia.	WHO surgical safety checklist	Postoperative mortality rates decreased from 1.2 to 0.92% OR 0.74, and length of admission decreased from 5.2 to 4.7 days. Reduction in mortality reached significance after 2–3 years. Independent of surgery duration.
Gama et al. 2019^19^	Brazil: 518 (control: 171, SSC: 347), Canada: 842 (control: 177, SSC: 665)	Canada and Brazil, university hospitals	Elective and urgent	Altered WHO surgical safety checklist (by each institution)	SSI rate decreased from 27.7%-25.9% in Canada and from 17.0%-14.4% in Brazil, not statistically significant. In Canada, no SSI in incomplete SSC and in Brazil 20% SSI in incomplete SSC, statistically significant difference.
GlobalSurg Collaborative 2019^20^	4843	76 countries	Emergency laparotomy	WHO surgical safety checklist	SSC associated with a lower 30-day perioperative mortality with OR 0.60, statistically significant. Greatest absolute benefit for emergency surgery in low- and middle-HDI countries.
Haugen et al 2019^34^	3702 (control: 1398, SSC: 2304)	Norway	Control: orthopedic 51.6%, thoracic 21.0%, neuro 27.5%, elective 49.6%, emergency 50.4%. SSC: orthopedic 67.6%, thoracic 17.0%, neuro 15.4%, elective 55.3%, emergency 44.7%.	WHO SSC	SSI decreased from 7.4–3.6% (OR 0.52). Antibiotics post incision decreased 12.5 to 9.8%, pre-incision increase from 54.5 to 63.1% and non-administration decreased 33–27.1%. Blood transfusion costs reduced by 40%.
Ramsay et al 2019^10^	6839736	Scotland	General surgery: pre-SSC 34.3%, SSC 31.7%, post-SSC 32.7%. Orthopedics: pre-SSC 15.3%, SSC 17.5%, post-SSC 17.6%. Other: pre-SSC 50.4%, SSC 50.8%, post-SSC 49.7%. Non-elective: pre-SSC 23.6%, SSC 18.8%, post-SSC 17.4%	WHO surgical safety checklist	Before SSC, inpatient mortality rate was 0.76%, after it was 0.46%. SSC associated with 36.6% reduction in mortality. Before, SSC mortality rates were decreasing by 0.003% per year, during implementation annual decrease was 0.069% and after 0.019%
Wang et al. 2019^47^	7209 (SSC: 3971, control: 3238)	China	Elective surgery to remove GI tumor: partial/ total gastrectomy, right/left hemicolectomy, Dixon, Hartmann, Miles, small bowel resection. General anesthesia 58.34% control and 79.93% SSC	WHO surgical safety checklist	The rates of morbidity and in-hospital mortality before and after SSC implementation were 16.43% vs 14.33% and 0.46% vs 0.18% respectively. Postoperative hospital stay in SSC group was shorter than that in control group (8 vs 9 days). SSC was an independent factor influencing postoperative complications (OR = 0.860).
Anderson et al 2018^81^	591	United States, children’s hospital	Pediatric surgery (burn dental, fetal, GI, OMFS, pulmonology and transplant surgeries)	WHO SSC	19% cases had 1 or more intraoperative delay (majority due to missing/malfunctioning equipment). No difference in adherence but increased fidelity for cases without delay (80.5% vs 77.1%)
Rodella et al 2018^48^	1166424	Italy, 48 public hospitals	MSK: 20.4– 22.2%, GI: 9.3– 11.6%, Ob/gyn: 7.3–8.8%, urinary: 6.9– 7.9%	WHO surgical safety checklist	Statistically significant differences between surgical interventions performed in hospitals with higher adherence to the checklist and in other hospitals with 30-days readmissions rate OR: 0.96 and LOS ≥ 8 days rate (OR: 0.88). No association with mortality,
Schmitt et al 2018^82^	80 (SSC: 40, control: 40)	Germany	OMFS procedures: routine dental extractions and biopsies, multiple extractions and osteotomies, routine/multiple implant placement and complicated implant placement and bone graft	Adapted WHO SSC (created by institution)	Statistically significantly higher frequency of incidents without the use of the checklist (n = 43) than with the use of the checklist (n = 10)
Shankar et al 2018^83^	1778	India, teaching hospital	Majority cases Ob/Gyn (223), general (226) and orthopedics (137). Some plastic surgery, pediatric surgery, urology, neurosurgery, dental. General anesthesia (626) and regional anesthesia (1152)	WHO surgical safety checklist	4.1% surgeries had complications with more than half being surgical wound infections. All patients received prophylactic antibiotics, SSC identified a deficit and corrected it in 27 patients
Westman et al 2018^84^	4678	Finland	Neurosurgery	WHO surgical safety checklist	Time from operation to infection shorter before than after checklist, effect in the onset of early HAIs. Overall incidence of SSIs of all patients did not differ at 4.1% vs 4.5%. No differences in superficial SSIs, deep SSIs, and deep organ SSIs.
Haynes et al 2017^85^	22514	United States, 14 hospitals (rural and urban, most were not teaching hospitals)	Adult inpatient surgery, obstetric excluded. Neurosurgery, head and neck, thoracic, cardiac, GI/abdominal, urology, gyn, ortho, vascular, skin/soft tissue	Adapted WHO SSC	Risk-adjusted 30-day mortality among SSC hospitals was 3.38% before SSC and 2.84% after, while mortality at other hospitals was 3.50% and 3.71% in those same years. There is a 22% difference between the groups on DID analysis.
Naidoo et al 2017^14^	3785	South Africa, 18 hospitals in public health sector	Maternal surgery consisting of CDs, laparotomies for ectopic pregnancies, uterine evacuations, removal of placentas and unplanned returns to OR	Modified World Health Organization surgical safety checklist for maternity care (MSSCL)	Significant improvements per 1000 patients in adverse incident rates (IRR 0.805), post op sepsis (IRR 0.619) and unscheduled return to OR (IRR 0.719). Greater reductions in maternal mortality in hospitals implementing MSSCL.
Anwer et al 2016^50^	3638	Pakistan	Elective surgery	WHO SSC	SSI in laparoscopic cholecystectomies was 20.8%, 13%, 5.68% and 1.12% in 1st, 2nd, 3rd and 4th year respectively as SSC use progressively increased from 20.4–89.9%.
Lacassie et al 2016^51^	58500	Chile	Emergency in 22.7% control and 23.5% SSC	WHO SSC	Mortality in hospital decreased from 0.82% before SSC to 0.65% after (OR 0.73). LOS also decreased from 3 days before to 2 days after.
García-París et al 2015^86^	134, control: 100, SSC: 34	Spain	Podiatric surgery: nail/skin surgery (66.4%), osteoarticular surgeries with implants (23.1%), osteoarticular surgeries without implants (10.4%).	WHO SSC	Statistically significant relationship between correct use of antibiotic prophylaxis and SSC use, reduction in LOS
Toor et al 2015^52^	613, control: 303, SSC: 310	United States	Similar rates for both control and SSC. Largest group was GI, 45 and 40%. Some hepatobiliary, gyn, urology, breast, skin cases.	WHO SSC	Optimal administration of antibiotic increased from 37.6 to 91% with SSC. Post-op infections decreased from 32.7 to 15.2%. LOS reduced from 7.8 to 6.5 mean
Baradaran Binazir et al 2015^53^		Iran		Modified WHO SSC	Complications pre-checklist 30% vs 12% post. Complications decreased by 58%
Kim et al 2015^54^	Long term follow up: 637, Short term follow up: 2106	Moldova, state general and trauma referral hospital	Similar cases for short and long term. Majority non-urgent, regional anesthesia, largest group general surgery (38.9% short vs 44.7% long term). Some OMFS, OB/Gyn, orthopedics, neurosurgery	WHO SSC, also implemented widespread use of pulse oximetry	Complication rate decreased 30.7%, SSI decreased 40.4%. Rate of hypoxemic events also decreased.
Lepänluoma et al 2015^55^	175, control: 103, SSC: 72	Finland	Neurosurgery	WHO SSC	Preventable complication requiring reoperations decreased from 3.3 to 2%. Mainly due to infection, 46% before and 39% after checklist. Infection related reoperations were 2.5% before vs 1.6% after. Adherence to checklist 78%
Helmio et al 2015^87^	223	Finland, tertiary, central, local and primary hospitals	ENT, 6.3% urgent	n/a	9.6% error in checklist item, 4.8% of injuries could have been prevented with properly used checklist
Biskup et al 2016^36^	Control: 2166, SSC:2310	United States	Plastics: 22% inpatient, 78% outpatient, 22% hand, 21% breast, 18% tegumentary, 13.5% head and neck, 10% aesthetic, 8% head & neck, 5% trunk, 1.5% micro, 1% LE	Modified WHO SSC (by surgeons at Loma Linda University Medical Center)	No significant decrease in complications (total or specific) for plastic surgery, found need for a more specific checklist
Chaudhary et al. 2015^64^	700, Control: 264, SSC: 271	India	GI surgery	WHO SSC with preoperative imaging and postoperative DVT modifications	Wound related, abdominal, and bleeding complications lower with checklist. High grade complications and mortality reduced. Number of complications per patient was higher for those with incomplete checklists than fully completed.
Haugen et al. 2015^56^	5295, control: 1305, SSC: 1671	Norway	Similar case distribution for control and SSC. Majority elective cases. Largest group was orthopedics (control 32.7%, SSC 55.3%). Other cases: thoracic, neurosurgery, general, urology.		Complication rates decreased from 19.9–11.5%, absolute risk reduction 8.4. SSC effect on complications significant with OR 1.95 even with adjustments for confounding factors. LOS decreased by 0.8. Mortality in hospital decreased from 1.9–0.2% in 1 out of 2 hospitals but overall, not significant.
Urbach et al 2014^88^	Control: 109341, SSC: 106370	Canada, all acute care hospitals in Ontario	Similar case distribution for both groups. Vast majority elective, majority outpatient. Mix of neuro, eye, ear, ENT, respiratory, CV, lymphatic, GI, GU, MSK, skin and breast.	CPSI, own design, WHO SSC	No significant reduction in mortality or complications. Risk of death 0.71% before SSC, 0.65% after. Risk of complications 3.86% before, 3.82% after implementation.
Boaz et al 2014^57^	760, Control: 380, SSC:380	Israel	Orthopedic	WHO SSC	Postoperative fever in 5.3% with vs 10.6% without checklist. 34% decrease in the rate of surgical wound infection after SSC.
Lepänluoma et al 2013^58^	Control: 83, SSC: 67	Finland	Neurosurgery	WHO SSC	Unplanned readmissions 25% vs 10% after checklist. Wound complications decreased from 19–8%. Consistency of documentation improved.
Kwok et al 2013^62^	Control: 2145, SSC: 2212	Moldova	General surgery, gynecology, neurosurgery, ophthalmology and oral-maxillofacial surgery, orthopedics. Control: urgent 50.5%, SSC: urgent 46.8%	WHO SSC	Complication rate decreased from 21.5 to 8.8%, infectious complications decreased from 17.7 to 6.7% and non-infectious from 2.6 to 1.5%, hypoxemic episodes decreased from 11.5 to 6.4%
Lubbeke et al 2013^15^	Control: 609, SSC: 1818	Switzerland, tertiary hospital	Control: 53% elective, SSC: 52% elective	WHO SSC (French version)	Unplanned return to OR in 7.4% before vs 6.0% after, RR 0.82; reoperation for SSI in 3.0% before vs 1.7% after, RR 0.56; unplanned admission to ICU in 2.8% before vs 2.6% after, RR 0.90; inhospital death in 4.3% before vs 5.9% after, RR 1.44. Checklist use during 77 cases prevented 1 reoperation for SSI
Tillman et al 2013^60^	Control: 10126, SSC: 9676	United States	Cardiac, colorectal, general, gyn, thoracic, vascular, orthopedic	WHO SSC (Scott and White version)	Significant reduction in patients with post-anesthesia care unit temperature < 98.6°F from 9.7–6.9%. SSI rates decreased from 3.13–2.96% overall, not significant. SSI rates similar for all services except colorectal surgery (24.1% vs 11.5%).
Rosenberg et al 2012^61^	Control: 212, SSC: 180	United States	Plastic surgery	Office-based surgical checklist (based on WHO SSC)	Total number of complications per 100 patients decreased from 15.1 to 2.72, absolute risk reduction 12.4. Site marking increased from 69.9–97.8%, complications decreased from 11.9 to 2.72%.
Bliss et al 2012^89^	Control: 246, SSC: 73	United States	Elective cases	WHO SSC	30-day morbidity: reduction in adverse event rates – 23.6% for control, 15.9% for team training, 8.2% for checklist use
van Klei et al 2012^59^	Total participants: 25,513; SSC: 11,151	Netherlands	Similar rates. Most frequent: 16.6% control vs 17% SSC emergency surgery, 18.3% control vs 17.3% SSC general surgery. Some CT surgery, neurosurgery, ENT, orthopedic, gynecology, plastics, vascular, eye surgery, dental and urology.	WHO SSC	Mortality decreased from 3.13 to 2.85% (OR 0.85) and related to checklist compliance. Full compliance association is 0.44 while association is 1.09 and 1.16 for partial and noncompliance
Yuan et al 2012^63^	Control: 232, SSC: 249	Liberia, 2 hospitals	Similar anesthesia for both groups. Majority general anesthesia (62.4% control, 54.6% SSC), some spinal, local, and conscious sedation. Control: 24.8% emergency general, 33.9% emergency OB, 29.1% other general, 12.2% other OB. SSC: 14.5% emergency general, 45.2% emergent OB, 21.8% other general, 18.5% other OB	WHO SSC	Introduction of checklist was significantly associated with reduced surgical site infections (adjusted OR: 0.28) and a reduced surgical complication (adjusted OR: 0.45). Association was significant only for Hospital 2 (OR: 0.12 and 0.35) and not for Hospital 1 (OR: 0.74 and 0.75)

## Data Availability

Literature review performed with materials from the University of Illinois library.
